# Two novel PCR-based assays for sexing of *Silene latifolia* and *Silene dioica* plants

**DOI:** 10.1016/j.mex.2024.102708

**Published:** 2024-04-11

**Authors:** Anna M. Hewett, Julia Sánchez Vilas, Frank Hailer

**Affiliations:** a School of Biosciences, Sir Martin Evans Building, Cardiff University, Cardiff CF10 3AX, UK; b Department of Ecology and Evolution, University of Lausanne (UNIL), Lausanne 1015, Switzerland; c Departamento de Bioloxía Funcional (Área de Ecoloxía) Facultade de Bioloxía, Universidade de Santiago de Compostelac/ Lope Gómez de Marzoa, s/n15782 Santiago de Compostela, Spain; d Cardiff University-Institute of Zoology Joint Laboratory for Biocomplexity Research, Beijing, China

**Keywords:** Dioecy, Sex markers, *Silene*, Spermidine synthase gene, PCR, PCR-based assays for sexing *Silene* plants

## Abstract

*Silene latifolia* and *S. dioica* are model systems in studies of plant reproduction, chromosome evolution and sexual dimorphism, but sexing of plants based on morphology is only possible from flowering stage onwards. Both species show homogametic females (XX) and heterogametic males (XY).

• Here we developed two assays (primer pairs *ss816* and *ss441*) for molecular sexing of *S. latifolia* and *S. dioica*, targeting length polymorphisms between the X and Y-linked copies of the spermidine synthase gene.

The two assays were successful in identifying known (flowering-stage) males and females from UK and Spanish populations, with an error rate of 3.1% (*ss816*; successful for both species) and 0% (*ss441*, only successful for *S. latifolia*). Our assays therefore represent novel tools for rapid, robust and simple determination of the genotypic sex of *S. latifolia* and *S. dioica*.

## Graphical abstract

**Figure:**
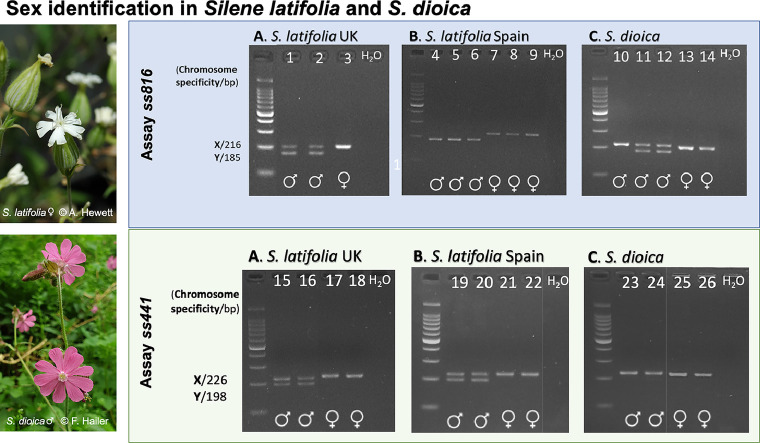

Specifications table

**Table:** 

Subject area:	Agricultural and Biological Sciences
More specific subject area:	Plant Science
Name of your method:	PCR-based assays for sexing *Silene* plants
Name and reference of original method:	HOBZA, R. and WIDMER, A. (2008), Efficient molecular sexing in dioecious *Silene latifolia* and *S. dioica* and paternity analysis in F1 hybrids. Molecular Ecology Resources, 8: 1274-1276. https://doi.org/10.1111/j.1755-0998.2008.02344.x
Resource availability:	Resources necessary are included in the text

**Method details**

## Background

*Silene latifolia* and *S. dioica* both display an active Y-system of sex determination, with heterogametic males (XY) and homogametic females (XX) [1]. Hence, the two species have become model systems for plant sex determination and sex chromosome evolution [[1], [2], [3]]. *Silene* species are also commonly used in studies of sexual dimorphism (e.g., [4,5]), although life history stages prior to flowering are rarely investigated [6]. Therefore, the ability to sex an individual plant already prior to flowering would e.g. enable studies of sex allocation and sex-specific physiological processes.

Unfortunately, existing molecular markers for sex determination in *Silene* are sub-optimal. Methods such as those in Zhang et al. [7] and Mulcahy et al. [8] often require post-PCR steps and lack additional fragments that amplify in both sexes to act as a control, potentially limiting their usefulness [9]. More recently, Hobza and Widmer [10] described a PCR-based method of sexing *S. latifolia* and *S. dioica*. However, we found this method to be unsuccessful in our laboratory when tested on UK populations, conceivably a result of inter-population differences on the sex chromosomes [11]. We therefore developed a novel molecular method for sex determination in *S. latifolia* and *S. dioica*, aiming to obtain a rapid, cheap and robust assay.

### Plant material

UK populations of S. *latifolia* and *S. dioica* were commercially ordered as seeds from Chiltern seeds (Wallingford, England, UK) then sowed and grown Cardiff University Talybont Glasshouses (51.500210, -3.200921). Spanish *S. latifolia* seeds were collected from 5 natural populations in Galicia, Northwest Spain (see [5]).

### DNA extraction

One ca. 75 mm^2^ disc of fresh leaf tissue was ground in 1.5 mL microcentrifuge tubes using micropestles. 400 µl of Edward's extraction buffer (200 mM Tris, 250 mM, 25 mM EDTA, 0.5% SDS) was added to ground samples. The tube was vortexed and incubated at 55 ⁰C for 10 min. The extract was centrifuged at 13,000 rpm for 1 min and 300 µl of the supernatant transferred into a sterilised Eppendorf tube. The supernatant was mixed with 300 µl 100% isopropanol and left at room temperature for 2 min. After centrifugation at 13,000 rpm for 15 min, the supernatant was removed and discarded and 300 µl of 70% ethanol was added to the pellet in the tube and left for 3 min. Following centrifugation at 13,000 rpm for 5 min the supernatant was removed and discarded, and the pellet was left to air dry for a few minutes. Finally, the pellet was dissolved in 100 µl of TE buffer (10 mM Tris, 0.1 mM EDTA), incubated at 55 ⁰C for 30 min, and stored in a freezer (-20 ⁰C) until required.

### Polymerase chain reaction (PCR)

Fragments were PCR amplified in 15 µl reaction volumes containing: 0.25U GoTaq G2 Flexi DNA polymerase (5 U/µl, Promega), 1x Promega Green GoTaq Flexi Buffer; 0.4 µM of each primer, 2.5 mM MgCl_2_, 167 µM of each dNTP along with 1 µl of DNA extract. PCR reactions started with 3 min at 95 ⁰C followed by cycling of 95 ⁰C for 30 s, 61 ⁰C (*ss816*) or 50 ⁰C (*ss441*) for 75 s, 72 ⁰C for 45 s, for 38 cycles, then a final 10-min extension step at 72 ⁰C. PCR products were run on 3% agarose gels at 120 V for 65 min, stained with SYBR™ Safe (ThermoFisher, UK) and viewed under UV light. Negative controls were included in each reaction to monitor for any contamination.

### Development of *Silene* sexing primers

Primer design was based on a sex-linked gene identified by Filatov [3] with differentiated copies on the X and Y chromosome: *S. latifolia* spermidine synthase gene (GenBank accessions: AY705437.1, AY705438.1; [3]). Two primer pairs (Table 1) were designed, targeting areas flanking insertion/deletion events of the X or Y chromosome sequences, resulting in the Y gene copy being visibly shorter on agarose gel than the X copy, for robust sex diagnosis [9].

**Table 1:** Details of primer pairs designed for sexing *Silene*. Primer name refers to its 5′ start position in the *S. latifolia* spermidine synthase DNA sequence from Genbank. Fragment length (bp) is the expected size in base pairs of the band for the Y and X copies.

Assay name	Primer name	Primer sequence (5′->3′)	Fragment length (bp)
*ss816*	816F1031R	CATGTTAGCCAACTCCAACGCTGAGAGGACAATCCAAAGTAGC	216 (X copy)185 (Y copy)
*ss441*	441F674R	TTTTTAAWATGGGGCGGTGMACTCCAATATAAGTATAGTGTAGA	226 (X copy)198 (Y copy)

## Method validation

**Fig. 1:**
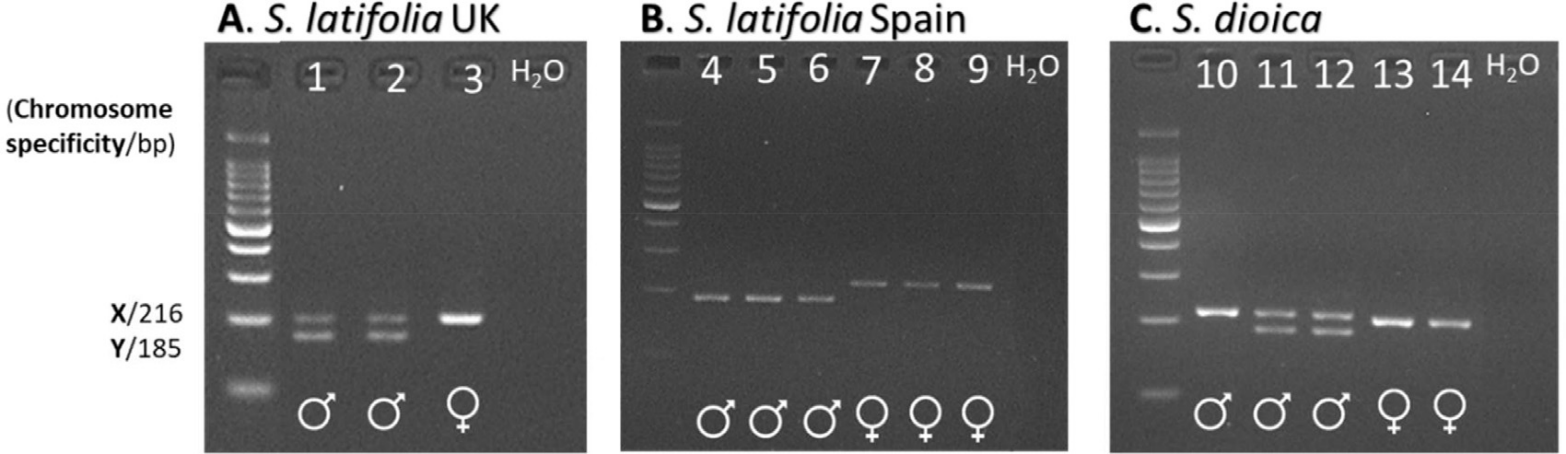
Sex identification results from assay *ss816*. A. *S. latifolia* UK populations (individuals 1–3) B. *S. latifolia* Spanish populations (4–9) and C. *S. dioica* (10–14). Note that in panel C, individual 10 (carrying male flowers) displayed the genotype of a female. H_2_O denotes the negative (no template) control.

**Fig. 2:**
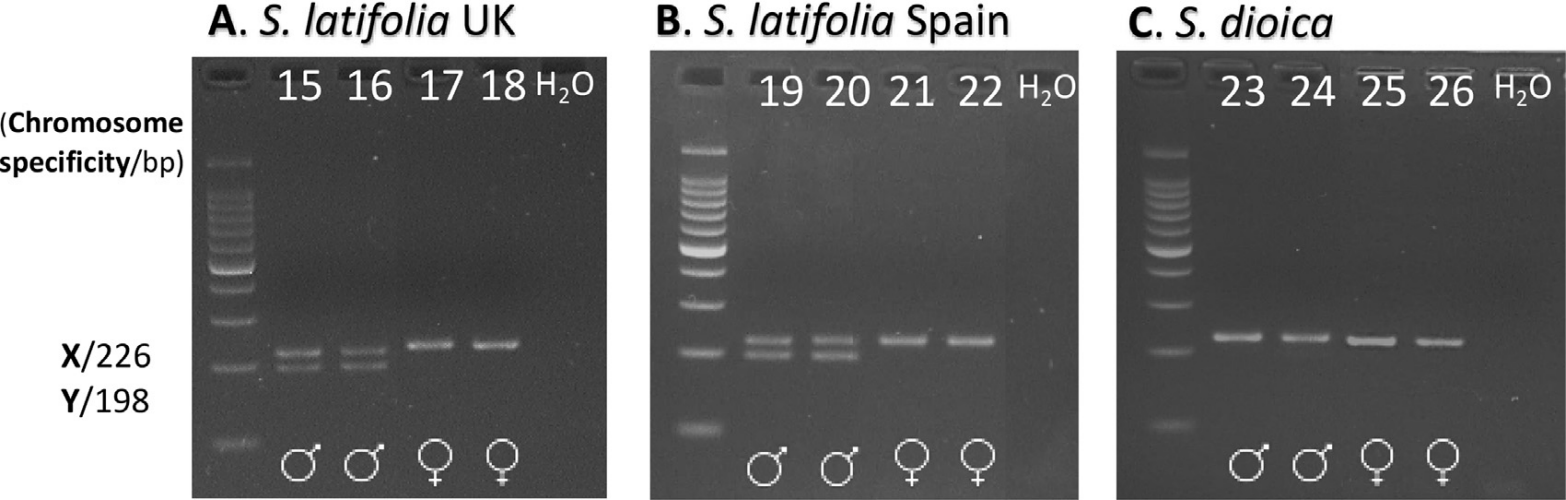
Sex identification results from assay *ss441*. A. *S. latifolia* UK populations (individuals 15–18) B. *S. latifolia* Spanish populations (19–22) and C. *S. dioica* (23–26). Panel C shows that ss441 was not successful in determining sex in *S. dioica*. H_2_O denotes the negative (no-template) control.

When applied to known-sex plants, both assays (*ss816* and *ss441*) generally provided the predicted outcomes for sexing: genotypic females displayed only the X-specific band, while genotypic males additionally showed the Y-specific band (Figs. 1A; 2A). When using assay *ss816* to sex 98 individuals, including samples from UK and Spanish populations of *S. latifolia* and UK populations of *S. dioica*, results matched the phenotypic sex in all but 3 cases (Table 2, and see below), corresponding to an error rate of 3.1%. While it does not affect the ability of accurate molecular sexing, it should be noted that in the Spanish population of *S. latifolia* the X-specific band only amplified in females, but not in males – still yielding the male-specific band in males, and the X band as an amplification in females (Fig. 1B). In addition, using *ss816* we obtained clear test results for 163 *S. latifolia* individuals, with the test providing non-ambiguous results as either male or female for each tested sample. However, since their phenotypic sex was unknown, this merely demonstrated that the assay gave clear, albeit unverified, results for a relatively large number of individuals. Use of *ss441* to sex 55 individuals from the same populations yielded assay results that matched the phenotypic sex without any mismatches, giving an error rate of 0% (Table 2). For this assay, *S. dioica* did not reveal discernible differences between males and females (Fig. 2C), leading to the inability to sex individuals. An additional 40 *S. latifolia* individuals with unknown phenotypic sex successfully amplified for *ss441*. Of these 40, 35 matched the sex predicted by assay *ss816*, and the remaining 5 were solely amplified by *ss441*.

**Table 2:** Summary of PCR amplifications of each primer pair assay. Column ‘Unknown sex’ indicates individuals with unknown phenotypic sex that were screened using our sexing assays. The error rate was calculated by dividing the number of unsuccessful/mismatched individuals by the total number of known-sex individuals tested.

	*ss816*				*ss441*			
	Male	Female	thereof mismatch	Unknown sex	Male	Female	thereof mismatch	Unknown sex
*S. latifolia* UK	28	37	2*	163	15	28	0	40
*S. latifolia* Spain	6	5	0	n/a	6	6	0	n/a
*S. dioica*	9	10	1*	n/a	0	0	0	n/a
Total	43	52	3		21	34	0	
Error rate			3.1%				0%	

⁎ phenotypic males that only amplified for the X band in the assay.

The three individual mismatches in *ss816* between the genotypic and phenotypic sex (two in *S. latifolia* and one in *S. dioica*) were all phenotypic males (exhibiting male flowers), yielding the assay outcome of a female. These same individuals did not amplify in the *ss441* assay. These results were confirmed by re-extracting DNA and repeating the PCRs. Contamination could not explain the outcome, since a Y band was missing, rather than an extra band occurring. The three observed mismatches do not necessarily discredit the accuracy of our assays, for three reasons. Firstly, the lack of clear amplification of *ss441* could suggest issues with the quality/quantity of the DNA extracts. Secondly, the target gene spermidine synthase is sex-linked, but not an area involved in sex determination [[3], [12], [13]]. Therefore, the expression of the phenotypic sex is independent of the presence of the region amplified by both primer pairs. Deletions in this gene could therefore explain our observations, consistent with the observed degeneration of *Silene latifolia* Y chromosomes [14]. Thirdly, it could also be suggested that the primer target sequence on the Y chromosome shows point mutation polymorphisms among populations [11,14], resulting in unsuccessful primer binding and failed amplification for some populations. Future work on sex chromosome polymorphism in *Silene* studies may reveal further details on the apparent mismatches between the sex inferred from flowers versus Y-chromosomal assays such as ours. Similarly, the observed relatively high inter-population differentiation for Y-linked genes in *S. latifolia* [11] could also explain why Spanish and UK populations of *S. latifolia* varied in assay suitability. It could be that the primers targeted an area of high mutation rates on the Y and X chromosomes leading to varied results of amplification, differing between and within species.

In conclusion, assay *ss816* successfully amplified the X and Y chromosome sequences of spermidine synthase in individuals of both *Silene* species, and *ss441* was successful for *S. latifolia*. Our assays can be used alone or in tandem to identify genotypic males and females of *S. latifolia* in UK and Spanish populations, as well as the related *S. dioica,* aiding ecological and genetic research. We anticipate that apparent ‘mismatches’ such as these found in the present study may in fact reveal interesting aspects of sex chromosome evolution in *Silene*.

## Ethics statements

We have used seeds and plants for our experiments, complying with our institution's safety and laboratory guidelines.

## CRediT authorship contribution statement

**Anna M. Hewett:** Validation, Investigation, Formal analysis, Writing – original draft. **Julia Sánchez Vilas:** Conceptualization, Methodology, Resources, Writing – review & editing, Supervision. **Frank Hailer:** Conceptualization, Methodology, Resources, Writing – review & editing, Supervision.

## Declaration of competing interest

The authors declare that they have no known competing financial interests or personal relationships that could have appeared to influence the work reported in this paper.

## Data Availability

Data will be made available on request.
